# IS ONLINE ACCESS AND ACTIVITY ASSOCIATED WITH BETTER MOOD IN OLDER ADULTS LIVING ALONE DURING THE COVID-19 PANDEMIC?

**DOI:** 10.1093/geroni/igac059.1474

**Published:** 2022-12-20

**Authors:** Yael Koren, Suzanne Leveille

**Affiliations:** University of Massachusetts Boston, Brookline, Massachusetts, United States; University of Massachusetts Boston, Boston, Massachusetts, United States

## Abstract

Older adults living alone during the COVID-19 pandemic face risks for social isolation and mental health problems. The goal of this study was to investigate the relationship between access to internet-capable devices or utilizing email/ texting, social networking [SN], or health-related internet use [HIU] and psychological outcomes (depressive and anxiety symptoms) among older adults living in the community during the early months of the pandemic, March, 2020-May, 2020. A cross-sectional analysis of the National Health and Aging Trends Study (NHATS, Round 10) was conducted, using multivariable logistic regression. Adults aged ≥85y were more likely to live alone than those aged 65-84y (34.3% and 24.9% respectively, p< 0.001). Additionally, 39.6% of women lived alone compared with 21.2% of men (p< 0.001). Fewer people living alone had access to an internet-enabled device than those living with others (10.4% and 5.4%, respectively). Among older adults living alone, utilizing SN and HIU was associated with lower likelihood of depression or anxiety (Depression: adj.OR 0.36, CI 0.17-0.77; Anxiety: adj.OR 0.31 CI 0.13-0.76). Alternatively, among those living with others, using email/ texting was associated with lower likelihood of depression and anxiety (Depression: adj.OR 0.23, CI 0.13-0.46, Anxiety: 0.33 CI, 0.17-0.67). In summary, internet activity was associated with reduced risk for depression and anxiety among older adults who lived alone or with others early in the pandemic. Further research is needed to better understand the potentially mitigating role of internet activities among older adults at risk for isolation and associated mood disturbances.

